# Augmented Reality-Based Real-Time Visualization for Structural Modal Identification

**DOI:** 10.3390/s24051609

**Published:** 2024-03-01

**Authors:** Elliott Carter, Micheal Sakr, Ayan Sadhu

**Affiliations:** 1 Department of Software Engineering, Western University, London, ON N6A 5B9, Canada; 2 Department of Civil and Environmental Engineering, Western University, London, ON N6A 5B9, Canada; 3 Department of Civil and Environmental Engineering, The Western Academy for Advanced Research, Western University, London, ON N6A 5B9, Canada

**Keywords:** augmented reality, structural health monitoring, real-time monitoring, modal analysis, system identification, application development

## Abstract

In the era of aging civil infrastructure and growing concerns about rapid structural deterioration due to climate change, the demand for real-time structural health monitoring (SHM) techniques has been predominant worldwide. Traditional SHM methods face challenges, including delays in processing acquired data from large structures, time-intensive dense instrumentation, and visualization of real-time structural information. To address these issues, this paper develops a novel real-time visualization method using Augmented Reality (AR) to enhance vibration-based onsite structural inspections. The proposed approach presents a visualization system designed for real-time fieldwork, enabling detailed multi-sensor analyses within the immersive environment of AR. Leveraging the remote connectivity of the AR device, real-time communication is established with an external database and Python library through a web server, expanding the analytical capabilities of data acquisition, and data processing, such as modal identification, and the resulting visualization of SHM information. The proposed system allows live visualization of time-domain, frequency-domain, and system identification information through AR. This paper provides an overview of the proposed technology and presents the results of a lab-scale experimental model. It is concluded that the proposed approach yields accurate processing of real-time data and visualization of system identification information by highlighting its potential to enhance efficiency and safety in SHM by integrating AR technology with real-world fieldwork.

## 1. Introduction

### 1.1. Motivation

As more of the world’s critical civil infrastructure nears the end of its usable lifespan, the urgency of rapid and real-time structural health monitoring (SHM) techniques becomes increasingly apparent. The American Infrastructure Report Card 2021 (ASCE 2021) highlighted the importance of protecting aging civil infrastructure, revealing that one-third of America’s infrastructure is vulnerable to rapid deterioration. Over the last several years, there has been significant development in SHM technologies to undertake robust, rapid, and remote structural inspection [1,2,3,4,5,6,7,8]. However, traditional SHM techniques face significant implementation challenges under adverse weather and busy operational conditions worldwide. These challenges include prolonged structural closures, dense instrumentation, the need to capture data from inaccessible areas, and the absence of synchronous data acquisition methods. Consequently, data collected by field inspectors or maintenance engineers during structural inspections often remains unprocessed for several days due to their presence in bulk and dense formats [9]. Furthermore, given the increasing prevalence of aging infrastructure worldwide and their spatial nature, the delayed processing of data prevents timely decision-making about the potential rehabilitation of structures. Therefore, there is a dire need for real-time visualization of condition data, such as operational modal analysis in the field of SHM [10,11]. In addition, as Industry 4.0 brings forth the technological advancements of modern times, linking SHM with Augmented Reality (AR) raises the field of civil engineering into evolved and more effective grounds. It provides real-time visualization of structural data, enhancing engineers’ ability to detect anomalies and make informed decisions promptly. AR overlays digital information onto physical structures, offering an intuitive interface for onsite inspection, maintenance, and repair, ultimately optimizing safety and extending the lifespan of infrastructure. The application, however, remains novel and open for improvement. This paper proposes a real-time visualization approach for remote and autonomous structural inspection by leveraging the capabilities of immersive sensing environments, such as AR. The proposed visualization framework was developed to provide field inspectors with a user-friendly and interactive interface, allowing for improved real-time diagnostic information on vibration data during onsite structural inspections.

The use of AR devices has been explored in a variety of fields. The AR system typically involves a camera and a display device such as a smartphone, a tablet, or a head-mounted device (HMD), which allows the developers to design dynamic holograms that can provide critical information for intelligent decision-making. Users can operate these headsets through gestures or voice commands, making virtual objects and interfaces interactable. In engineering, ref. [12] portrayed the vast areas of knowledge implementing AR, such as electronics, construction, robotics [13], and sustainability [14]. Civil and structural engineering, more specifically, has embarked on the path of integrating AR, yet its focus has mainly been on the infrastructure’s construction phase [15,16]. Placing a widely adopted industrial tool in context, information models from building information modeling (BIM) technology are utilized as database models for sending information to be visualized in AR. BIM is widely used in the architecture, engineering, and construction industry to improve the design, construction, and operation of infrastructure and has been widely used in reviewed applications. These models include information about the structure’s geometry, spatial relationships, geographic information, quantities, and properties of structural components [17]. Ref. [18] developed an AR interface for mobile devices based on a BIM system. A 3D model was first developed and linked to the IFC format. The model was then exported to an OBJ file format for executing the AR system in the Layar mobile application. The schedule of construction was compared with the actual situation on site using a color-coded AR visualization. Ref. [19] developed an application for transferring BIM components to a real-life AR interface. Users could observe real-life views through an iPad with real objects highlighted in AR using 3D geometry, depending on the BIM model. Ref. [20] discussed the use of AR to view the model in static, panoramic views solely for architectural purposes. They explored software that did not support AR viewing modes and had very limited interaction with digital models.

### 1.2. Development of AR as a Sensor in Operation and Maintenance

Throughout the literature, several other case implementations have utilized AR devices as a sensory means of inspection while relaying valuable information about deficiencies present in the infrastructure. These studies exploit AR’s technological specifications, such as the camera of HMDs, to retain parametric data tracing structural damage. Ref. [21], for example, discussed the integration of AR with concrete bridge inspection. The discussion introduces a theoretical approach to using a sensory AR system to feed an information model. In another study, ref. [22] developed a crack inspection system using a head-mounted AR device and a high-resolution camera. The camera first captured the concrete surface, applied crack detection, and then projected the crack-detected image into the AR display captured by the HMD for color-coded crack width measurement. [16] discussed the implementation of an AR application that measures the moving distance of an object, a process useful for SHM inspections. It invoked virtual images to mark the object’s original position, allowing researchers to measure the distance between the original and current positions of the object. Ref. [23] developed an inspection methodology using an AR headset that quantitatively documented irregularly shaped sidewalks, measured the length and geometry of cracks in concrete, and proposed a framework for developing AR-based applications for structural inspection.

Ref. [24] deployed an application using Unity to identify the type of damage on concrete surfaces using an AR interface. The framework also enabled geometry measurements of the cracked surfaces, leveraging inspection in hard-to-reach locations. An ML model was deployed to the cloud until it was called by Unity for app deployment in the AR glasses. Ref. [25] explored the possibility of connecting infrared thermography with AR to enhance the visualization of SHM information. With Unity, a primary tool in developing HL applications, a user interface was developed using the main camera, allowing users to look around their virtual environment and thermal views were shown as 3D objects in the virtual Unity environment. Ref. [26] developed a computer vision algorithm combined with AR to localize fatigue cracks in structures that might easily be overlooked due to their small size. The result of this approach led to an AR-based automatic fatigue crack detection and localization approach that can provide holograms overlaid during onsite visual inspections with near-real-time results.

### 1.3. Development of AR in Operation and Maintenance via Visualization in BIM

In phases of operation and maintenance, SHM adheres to the requisites of preserving infrastructure serviceability. The use of AR as an appended tool ameliorates the process of strategic maintenance while offering user-friendly and easy-access vital structural information [15]. Ref. [27], for instance, developed a framework for linking environmental data to an AR visual interface while incorporating BIM and sensor data. The acquired data was integrated into a BIM model. A game environment subsequently called for the geometry, sensor, and location data to be presented in an AR interface on a mobile device using virtual charts. Ref. [28] developed a methodology for adopting BIM integration in an AR interface. The study incorporated the use of Unity3D for building the AR application and an interface to calibrate the physical and digital models. The system aided in facility management and acted as an inspection tool that is powered by back-end processes stored on an offline server. Ref. [29] developed an inspection method using drone-based photogrammetry, BIM, and AR. The users synchronized the visualization of the BIM model with aerial video using a coordinate transformation algorithm. The framework finally permitted data uploading, AR inspection, querying historical records, and configuring the system’s settings. AR annotations, such as inspection specifications, safety issues, and reports, were visualized in user-friendly interactive interfaces over the BIM model. Ref. [30] built upon finite element analysis to build an SHM system using IoT sensors on a bridge. An AR-3D visualization application was built to overlay the IoT data with the help of BIM-based knowledge.

### 1.4. Development of AR in Operation and Maintenance via Visualization in Other Information Models

Information models, however, are not only limited to BIM for extending the application to AR visualization. Others have employed cyber-physical systems, such as a digital twin (DT), to serve as a liaison between the physical world and the information visualized in AR. A DT is a digital replica that mirrors the real-world counterpart and is used for various purposes, including monitoring, analysis, simulation, and control. DTs leverage data from sensors, Internet of Things (IoT) devices, and other sources to create a near-real-time digital model that reflects the current state and behavior of the physical entity [31]. Ref. [32] developed a framework for visualizing a DT using an AR system. The user interface acts as an independent web browser. Web services enable users to retrieve information requested from the available resources. The resources are retrieved from databases using querying protocols such as structured query language (SQL), hypertext transfer protocol (HTTP), and others. Ref. [33] developed a framework for visualizing the strain data of mechanical equipment using a DT and IoT. Strain sensors connected to an Arduino Mega transfer data via Wi-Fi to an IoT server. An in-built toolbox carried the analysis online until the AR glasses requested the visualization of the information when a QR code was scanned. Ref. [34] created a DT system to maintain a bridge using AR technology. The users integrated static data from inspections, historical data, and damage records with dynamic data from real-time sensor monitoring to create the DT. Azure IoT hub and Azure SignIR service were used to connect the dynamic data with BIM, while Unity 3D was used to link the DT with AR technology.

### 1.5. Development of AR in Operation and Maintenance via Visualization without Using Any Information Models

The translation of information models, however, into an AR platform demands high computational processing, a characteristic that is absent in portable AR devices such as HMDs. Autonomously self-updating a model with densely cumulated information about a large infrastructure’s components remains a major obstacle for real-time monitoring. Therefore, utilizing a 3D virtual model is disregarded in this study to fully comprehend the real-time monitoring process without jeopardizing computation power. Along this line of study of disregarding 3D modeling, ref. [35] proposed a method based on Virtual Tours for representing SHM data. The users documented their structure using connected spherical panoramas to facilitate 3D comprehension. The relevant data in the form of images, text, and files were linked to the environment and accessed by the user through an interactive, user-friendly interface. Images created the 3D representation instead of 3D models that require high computation and storage requirements. Ref. [36] integrated image-based documentation and AR for visualizing SHM data in the built environment. The methodology captured an image, enabled annotations on the target’s point cloud, and projected the annotations back into the 2D image for off-site and onsite inspection and viewing. Ref. [37] developed a connection between AR and a wireless strain sensor. The study established an IoT sensor that fed data to a MySQL server. The data were linked to an AR device where real-time data was visualized over the physical target. In another study, ref. [38] developed a human-centered interface that provides workers with real-time access to structural data during inspection and monitoring through an AR environment. The interface displayed real-time experiment data of maximum displacements, displacement time histories, and easy access to manipulation of the experiment data. In those studies, however, no form of raw data processing or modal analysis was achieved within AR for real-time diagnostics of the acquired data. Other studies, such as [39], utilized domain adaptation to synchronize and convert the damage-sensitive natural frequencies into a unified feature domain. A k-nearest neighbors model was then trained to understand the health status of the original dataset and subsequently anticipate forthcoming instances obtained from continuous monitoring. Nevertheless, its implementation with AR and real-time visualization is not addressed. Therefore, this paper proposes a novel and comprehensive methodology for visualizing real-time SHM data, including time-domain, frequency-domain, and system identification information such as natural frequencies and mode shape of a dynamical system using AR.

The paper is organized as follows: First, a brief background of the selected AR device, the Microsoft HoloLens 2 (HL2) is provided. Then, the proposed AR methodology is presented, including the overall workflow of the system, the application development, the web server communications, and the means of displaying experiment data in real-time through the HL2. The results of the data visualization are presented next using a lab-scale experimental model. Finally, the conclusion section summarizes the key findings, limitations, and future work of the proposed research.

## 2. Description of the Selected AR Device

**Figure 1:**
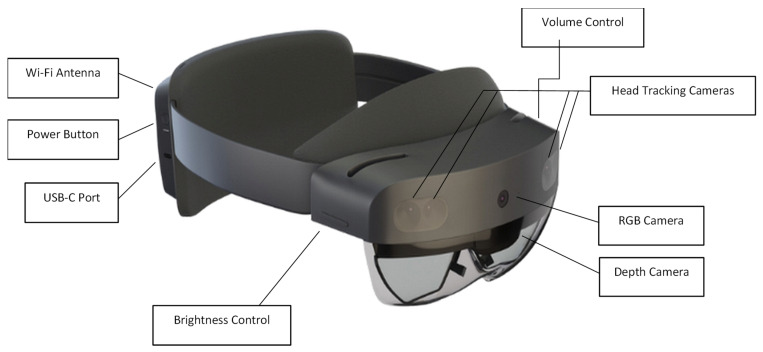
The basic hardware of Microsoft HoloLens 2.

In this paper, the capabilities of the Microsoft HL2 headset are leveraged to develop the proposed real-time structural inspection technology. HL2 runs on the Windows Holographic environment, which is an operating system based on the design of Windows 10, providing users with a performant, secure, and familiar platform. This presents itself as an ideal tool for fieldwork, seamlessly fitting into SHM contexts by leaving users free to view their surroundings within AR environments and use both hands. The device’s high-resolution image sensing and expansive field of view allow inspectors to immerse themselves in AR environments and perform analysis on the acquired data from within. The HL2’s built-in depth sensor allows virtual objects and text to be positioned precisely within real-world coordinates, enhancing the overall inspection experience. To further innovate this foundation, the HL2’s Wi-Fi capabilities are used to facilitate seamless data transmission between the headset and an external web server. The hardware of the HL2 and some of its key components are highlighted in Figure 1.

## 3. The Proposed AR-Based SHM Methodology

This research proposes new advancements in using AR to diagnose structural inspection data in real-time. The proposed approach consists of three steps. The first step begins with developing an AR application in the Unity engine that provides users with an intuitive platform for reading and interpreting experiment data retrieved from a database. Secondly, a server is set up to host a database for storing experiment data and managing external web requests. During experiments, data transmission between the server and HL2 is facilitated through HTTP requests. Thirdly, the AR application runs on the HL2 processes and visually represents the retrieved data, projecting it within the mixed reality environment in real-time. This allows for immediate and interactive structural health inspections within the physical context.

**Figure 2:**
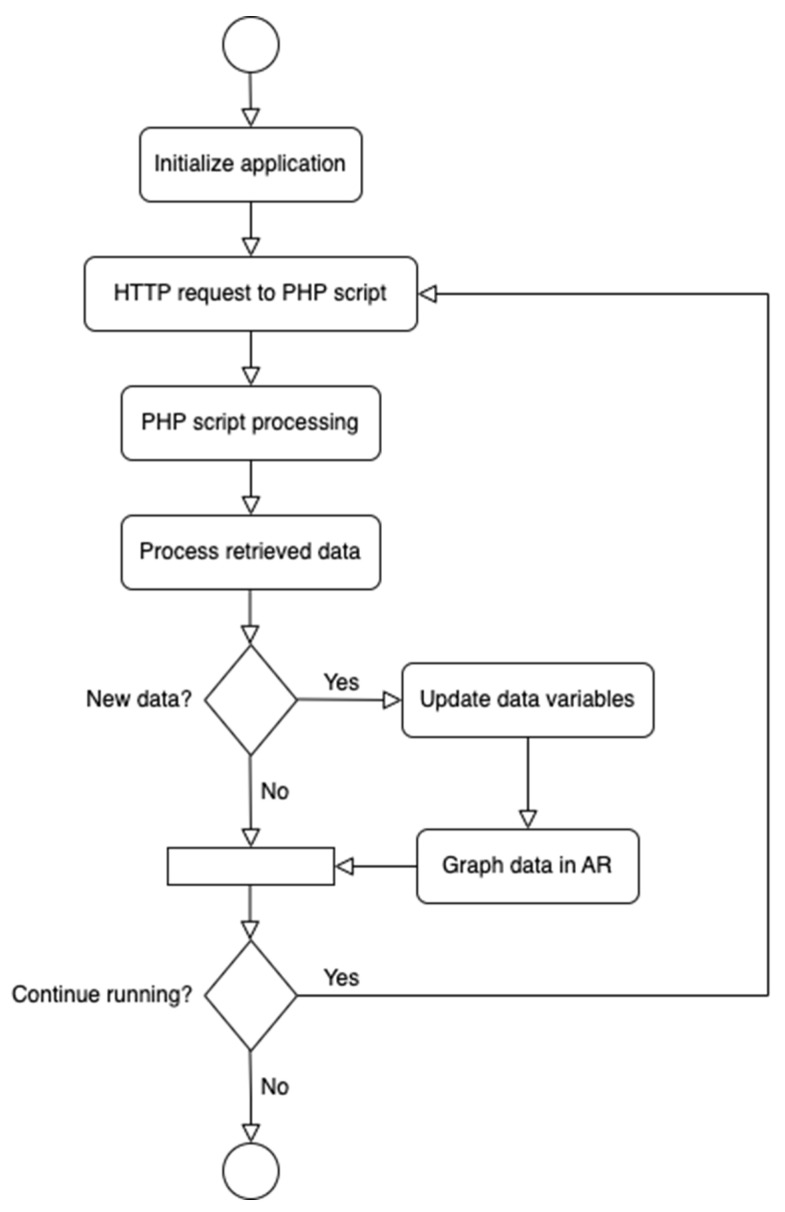
Flowchart of the proposed methodology.

**Figure 3:**
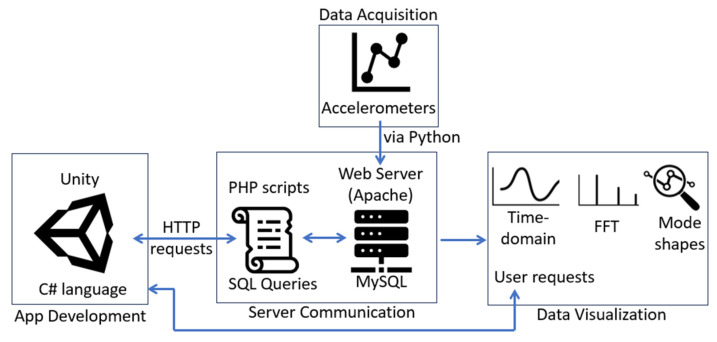
Relationship and communications between three modules of the proposed methodology.

Figure 2 shows a flowchart of the proposed data retrieval system. Once deployed to an HL2 model, users can send HTTP requests through the application to execute Hypertext Preprocessor (PHP) scripts on the server via Wi-Fi. The PHP scripts used in this research are responsible for querying the MySQL database and echoing retrieved data back to the Unity application. This enables the effective visualization of real-time experiment data within the AR environment and encompasses additional functionalities such as frequency-domain visualization and modal property estimations from the output data. This paper provides an overview of the development and authentication process of this new application. A deeper dive into the separate modules of the framework (i.e., application development, server communication, and data visualization) is subsequently explored. Figure 3 highlights the relationship and flow between the individual modules.

### 3.1. Application Development

The AR application is developed in Unity, and the scripts are written in C# through Visual Studio. To appropriately configure the application for AR, an AR Toolkit (Microsoft 2023) [40] is deployed, which is an open-source software development kit distributed by Microsoft. The Unity application is developed with Universal Windows Platform support, allowing deployment to a HL2 device. Within the application, users can use virtual buttons located throughout the holographic interface to retrieve, visualize, and perform operations on collected experiment data. These buttons can be activated through either hand gestures or speech commands, depending on the use case.

### 3.2. Server Communication for Remote Data Retrieval

In this research, XAMPP, a free and open-source cross-platform development environment, is deployed. The software that comes pre-installed with XAMPP (version 8.1.17) includes Apache and MySQL. Apache is a widely used web server software responsible for local HTTP request and response handling within the local development environment. MySQL is an open-source relational database management system that is very popular worldwide due to its efficiency and accessibility and, as a result, is chosen for storing experiment data for this research. In this study, the corresponding database table contains four columns: one for the base excitation and three for the recorded acceleration channels of the experimental model. However, the number of columns can be customized depending on the use case requirement. Once the time-domain data is acquired for all channels of measurement, they are processed using frequency-domain transformation followed by modal identification. 

### 3.3. System Identification within the AR Environment

It is important to note the existence of several operational modal analysis algorithms present within Python [41,42] The modal identification process in this study implements the use of CESSIPy (Civil Engineer Stochastic System Identification for Python), an open-source module developed by [43] and has proven effective and successfully fitting the experimental case. CESSIPy is implemented in Python (version 3.9) and requires ARPy, a package freely available from GitHub, and the popular libraries are NumPy, Matplotlib, and SciPy. The module estimates the eigen frequencies, damping ratios, and modal shapes from a data set of acceleration measurements using five different methods of Stochastic Subspace Identification (SSI). In this module, three time-domain methods are available: Covariance-Driven SSI (SSI COV), Data-Driven SSI (SSI DATA), and the instrumental variable method (IV). Additionally, two frequency-domain methods are available: Basic Frequency Domain (BFD) and Enhanced Frequency-Domain Decomposition (EFDD). For this study, the SSI COV method [44] was employed within this application. Ref. [45] were also successfully inspired by the SSI COV to build an automatic operational modal analysis system. It was described as an ML technique (random forest) to control the parameters of SSI COV to execute modal estimates. The following steps are implemented within the selected version of the SSI method [44] used in this study.

1. Model Equations: The state-space model is given by:

*x_k_*_+1_ = ***A****x_k_* + *w_k_*

*y_k_* = ***C****x_k_* + *z_k_*

where:

*x_k_* is the state vector of dimension *n* at time *k*.

*w_k_* is the process noise due to disturbances and modeling inaccuracies.

*y_k_* is the output vector.

*z_k_* is the measurement noise.

***A*** is the state matrix.

***C*** is the observation matrix.

2. Estimation of Model Matrices **A** and **C**:
  - The model matrices **A** and **C** are estimated from the output covariance matrix.
3. Eigenvalues and Eigenvectors:
  - Calculate the eigenvalue matrix *Λ_d_* and the eigenvector matrix *Ψ* of ***A*** using eigenvalue decomposition. *Λ_d_* is a diagonal matrix with the discrete eigenvalues *μ_i_*.
  - Calculate continuous-time eigenvalues *λ_i_* from *μ_i_* using the sampling time Δ*t*:
    *λ_i_* = *ln*(*μ_i_*)/Δ*t*
  - Calculate the eigenfrequencies *f_i_* from the continuous-time eigenvalues:
    *f_i_* = |*λ_i_*|/(2π)
  - Calculate the damping ratios *ζ_i_* from the real and absolute parts of the continuous-time eigenvalues:
    *ζ_i_* = *real*(*λ_i_*)/|*λ_i_*|

4. Mode Shapes:

The mode shapes *ϕ_i_* are the columns of the matrix *V = CΨ*, where *Ψ* is the eigenvector matrix of ***A***:

*V =* [*ϕ*_1_, …, *ϕ_i_*, …, *ϕ_n_*]

The data collected from the accelerometers is automatically written to a text file with a specified path name on the server. To facilitate the seamless transfer of data to the MySQL database, a Python script named ‘SensorToDatabase.py’ is written. This script periodically monitors the text file and attempts to upload new data to the database at intervals of approximately three seconds. This functionality is achieved by implementing a variable, ‘last_processed_line’, which keeps track of the last processed line in the text file. During each loop, the script compares the length of the text file in rows to the value of ‘last_processed_line’, preventing duplicate data from being uploaded. If the values are equal, no new data is added to the database, and no actions are performed. However, if the length of the text file exceeds ‘last_processed_line’, all new lines of data are stored in the MySQL database, and the value of ‘last_processed_line’ is updated to match the length of the text file. When the database table is cleared, the value of ‘last_processed_line’ is reset to zero to ensure accurate data uploads for subsequent experiments. Communication between the HL2 application and the server was established through PHP and SQL queries, enabling access to data stored within the MySQL database on the server. The PHP scripts used in this experiment, along with their functionalities, are outlined in Table 1.

### 3.4. Data Visualization 

**Figure 4:**
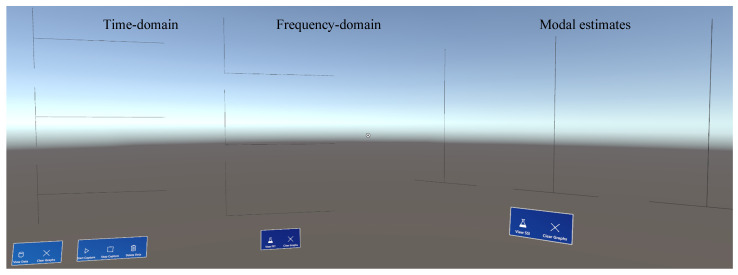
Full view of the blank initial interface in Unity simulation.

When the application is launched, the user is presented with an interface consisting of several empty (i.e., placeholder) plots. The plots are world-locked, ensuring their positions remain fixed regardless of the user’s relative position. The interface consists of three sections, each for plotting different properties of a dynamic experiment: time-domain data, Fast Fourier Transform (FFT), and mode shape estimation. Each section contains multiple subplots and controls for visualization and data-related operations. Figure 4 shows the default view of these windows upon starting the application.

**Figure 5:**
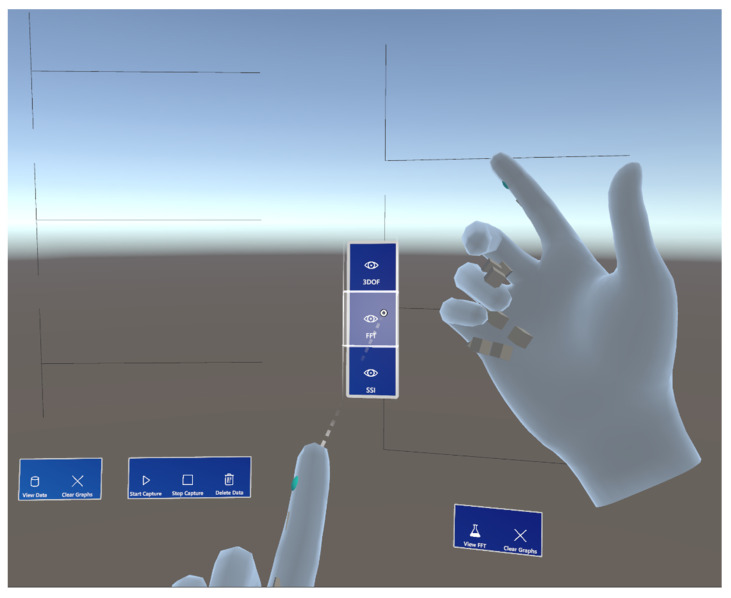
Result of using the hand menu to reveal the system identification information.

The visibility and positioning of each window can be toggled using a hand menu at any time, as demonstrated in Figure 5. This can be useful for changing experiment locations within a single session or hiding windows with unneeded information, for example. This system allows for a quick and intuitive way for users to set up their holographic environments according to their preferences.

#### 3.4.1. Visualization of Time-Domain Information

**Figure 6:**
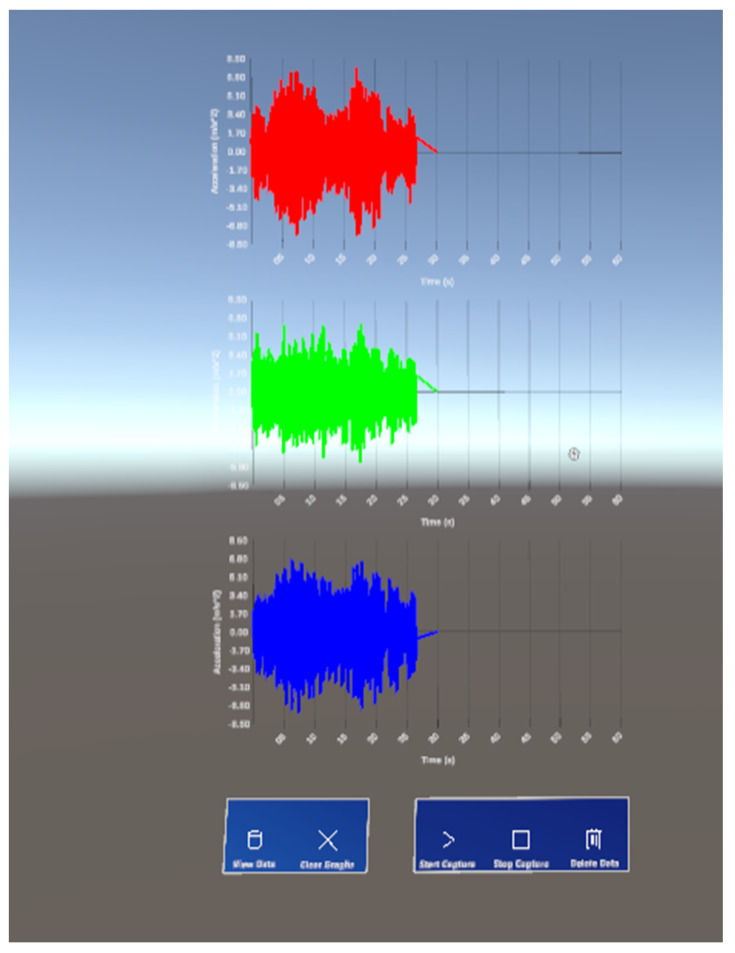
Using the “View Data” button to show time-domain data within the Unity test scene.

The time-domain interface contains five buttons: ‘Get Data’, ‘Clear Graphs’, ‘Start Capture’, ‘Stop Capture’ and ‘Delete Data’, each with specific functionalities detailed in Table 2. Figure 6 demonstrates the real-time visualization aspect of how acceleration data from all three sensors is graphed in parallel as sample data is read from the MySQL database.

#### 3.4.2. Visualization of Frequency-Domain Information

The frequency-domain visualization window is responsible for calculating and displaying the FFT of the acquired time-series data shown in Figure 6. This is achieved by using the open-source ‘Math.NET’ numeric library in Unity to perform forward Fourier transforms on all three sensor streams before plotting the results. The frequency-domain visualization interface contains two buttons: ‘View FFT’ and ‘Clear Graphs’, each with functionalities detailed in Table 3. HL2 is able to calculate and display frequency-domain information from locally stored sample data upon user prompt graphed in the mid-section of Figure 4.

#### 3.4.3. Visualization of System Identification Information

The stable mode shapes estimation window is responsible for communicating with the CESSIPy Python library, which employs SSI techniques to identify eigenfrequencies, damping ratios, and mode shapes from output-only data. This is achieved by estimating the matrices from the SSI COV method. Similar to the frequency-domain visualization window, this interface contains two buttons: ‘View SSI’ and ‘Clear Graphs’, as detailed in Table 4. The HL2 device finally displays mode shape estimations from sample acceleration data using CESSIPy- graphed in the right-most section of Figure 4.

## 4. Results and Discussion

### 4.1. Experimental Setup

**Figure 7:**
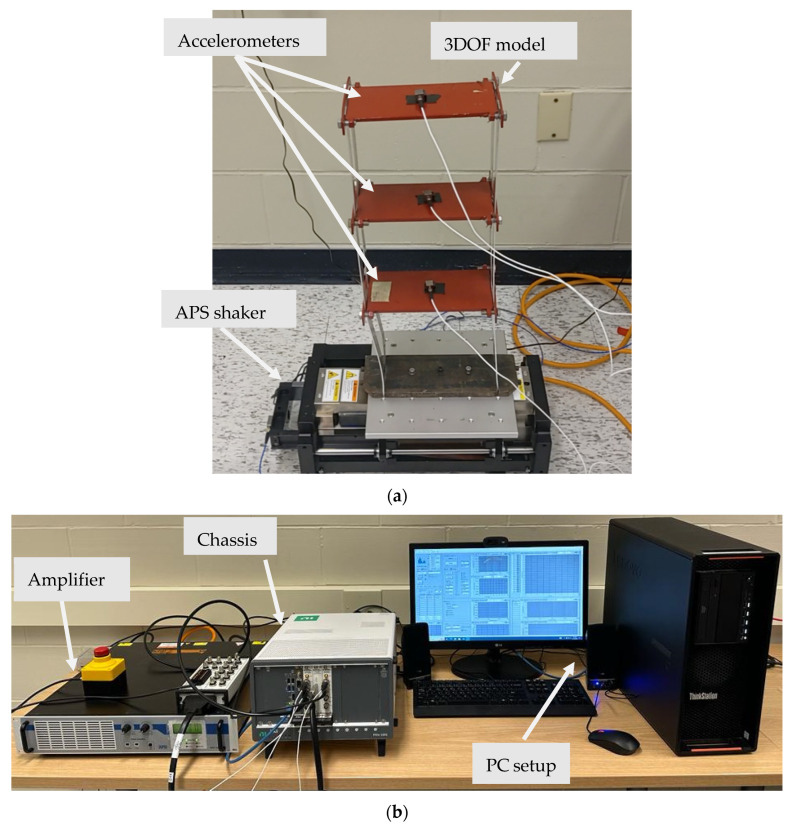
The experimental setup: (**a**) 3DOF model (**b**) DAQ chassis system.

To test the performance of the application running on a HL2 device in real-time, a three-degree-of-freedom (DOF) model is mounted on a shake table (APS 113 manufactured by APS Dynamics, San Juan Capistrano, CA, USA). Each story measures 200 mm in height and 250 mm in width. The story slabs have a thickness of 10 mm and are supported by stainless steel columns of 40 mm × 3 mm. The APS 113 is a long-stroke shaker with linear ball bearings. The output excitation signal is regulated via its power amplifier. The data acquisition system is composed of the PXIe-1092 chassis (manufacturer: National Instruments, Austin, TX, USA), which manages the input and output signals. A Lenovo ThinkStation PC (3.70 Ghz@4.50 Ghz, 32 GB Ram, 1.0 TB SSD, Nvidia Geforce, Santa Clara, CA, USA) hosts the software that manages the input and output signals. Three wired accelerometers (i.e., sensitivity (±10%): 1000 mV/g, range: ±5 g pk) are securely attached at each story level of the structure. A white noise random excitation ranging from 1 to 100 Hz with an acceleration root mean square of 0.5 m/s^2^ is executed. Figure 7a sums up the setup of the mounted structure on the shaker, while Figure 7b represents the entire Data Acquisition (DAQ) chassis system.

### 4.2. Application of Proposed AR-Based Visualization

**Figure 8:**
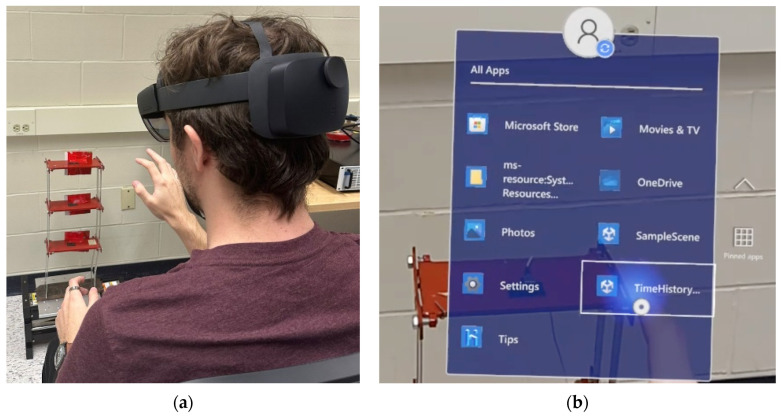
(**a**) Tracking the targeted structure using HL2 and (**b**) accessing the AR application of time-domain information.

**Figure 9:**
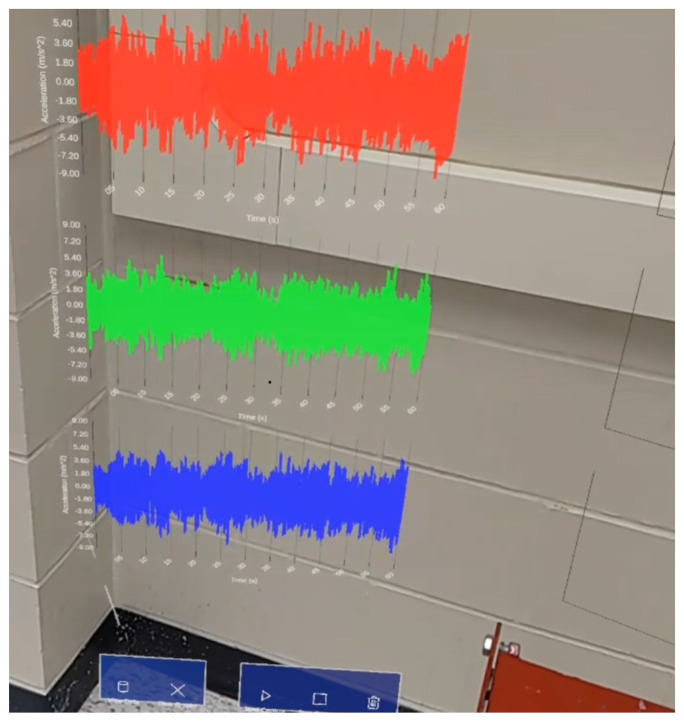
Capture of real-time acceleration time history.

**Figure 10:**
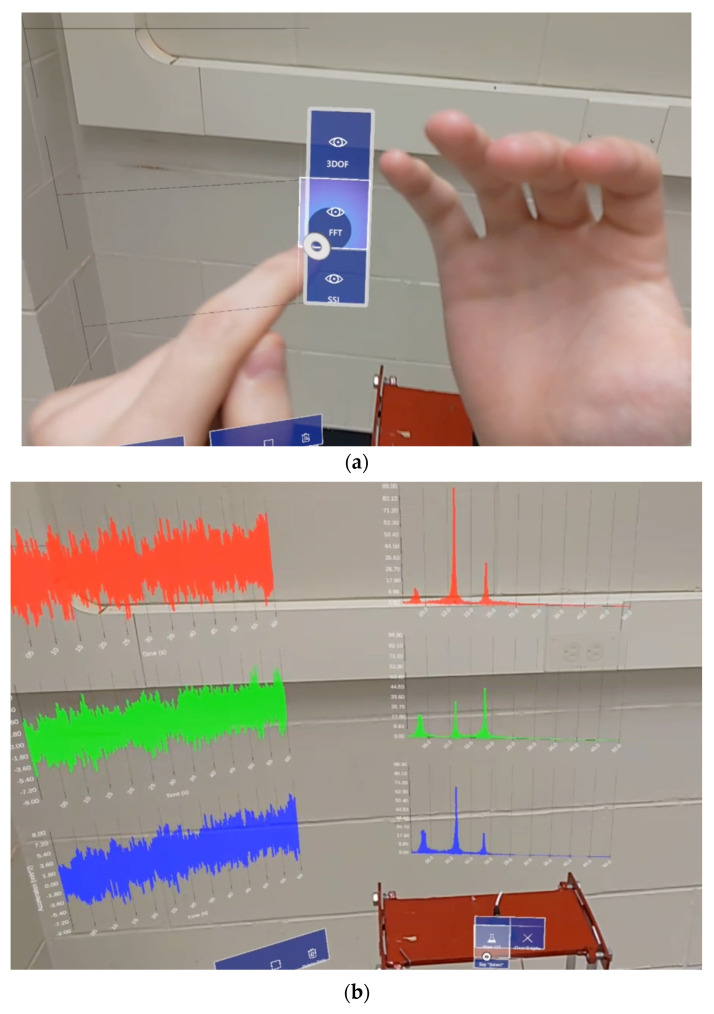
(**a**) Accessing the FFT feature and the (**b**) capture of real-time frequency-domain information.

**Figure 11:**
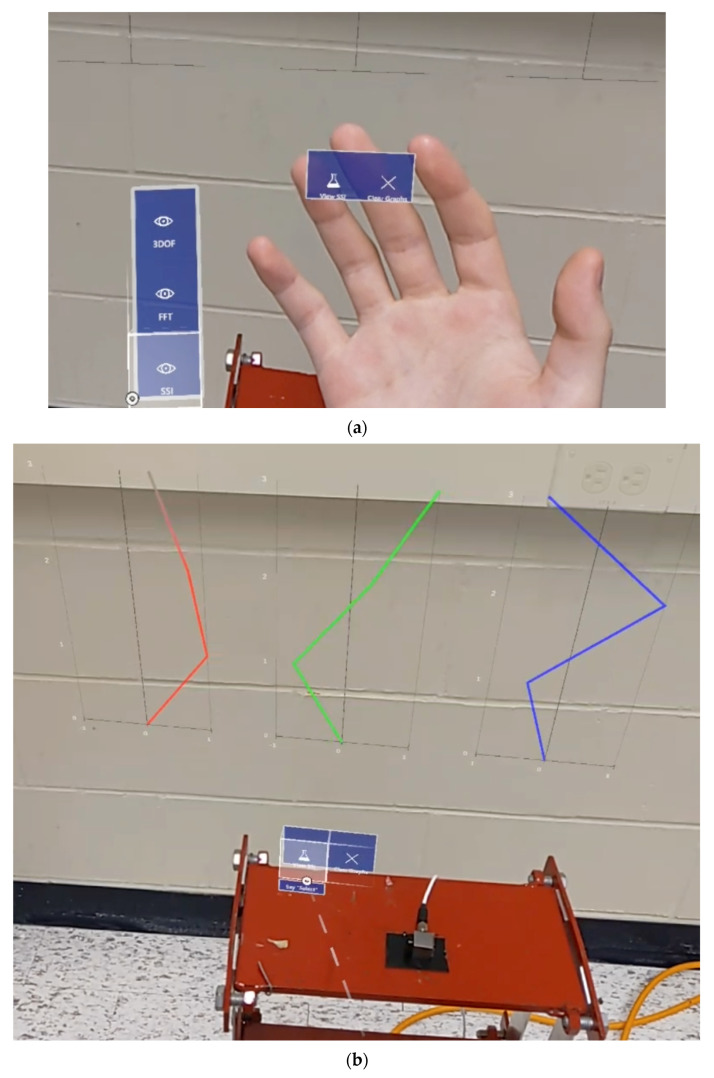
(**a**) Accessing the mode shape feature and the (**b**) capture of real-time modal identification information.

While the shake table is initialized, the “Start Capture” feature is activated from within the AR application to check the data file for new acceleration data. Because the accelerometers capture data in voltage, the data must first be converted to acceleration at a rate of 106.0 mV per m/s^2^ before being processed by the Unity app. Via three sensors, the time-history data is seamlessly streamed to the HL2 device and plotted in parallel. The data is collected for 60 s at a frequency of 100 Hz, resulting in 6000 active plotted data points per channel without any noticeable performance drops. The virtual graphs are constantly updated throughout the inspection period. The system allows the observation and analysis of modal information over a continuous data collection period while standing directly over the targeted structure, as represented in Figure 8a. Figure 8b shows the user accessing the developed application (TimeHistorySHM) on the HL2 system. The live visualization of the updated charts characterizes the system as a real-time monitoring process while securing a comprehensive modal analysis and system identification strategy. In addition, the system has the capability of displaying the last updated versions of the time-domain graphs, as seen in Figure 9, if any temporary manual or technical interruptions in the connection occur. Subsequent interfaces are used to calculate and display the frequency domain, as shown in Figure 10, in addition to the mode shape estimation using SSI, highlighted in Figure 11, which is estimated using the proposed application.

**Figure 12:**
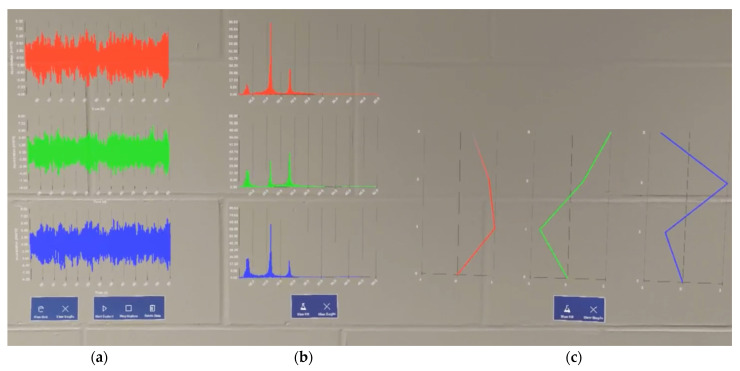
Capture of complete real-time graph generations for (**a**) time-domain data, (**b**) frequency-domain data, and (**c**) mode shape estimations.

The end visualization display strives to achieve a monitoring process under the structure’s normal behavioral conditions. It can be observed that the proposed tool can visualize all the essential diagnostics information associated with SHM data, as shown in Figure 12, which may be useful for the maintenance engineer or inspector onsite. 

## 5. Conclusions

In this paper, a visualization framework is developed for real-time structural inspection data using AR. The proposed visualization interface consists of three main sections: time-domain visualization, frequency-domain visualization, and mode-shape visualization. The time-domain visualization window allows the user to visualize real-time structural experiment data from a remote database via Wi-Fi. Subsequently, the frequency-domain visualization window can be used to natively compute and visualize Fourier transforms on the acquired time-series data. The mode shape estimations are obtained using CESSIPy, a Python library for stochastic system identification in civil engineering. To facilitate communication between the HL2 device and the server, PHP scripts are used. All PHP and Python scripts are contained in a local XAMPP development environment, which includes an Apache web server for processing HTTP requests and a MySQL database for storing live experiment data. These resources are accessed on the HL2 device through a Unity app via HTTP requests. 

The accuracy of the module used in this research was assessed by performing a shake table experiment and visualizing its time domain and frequency domain information in MATLAB. The results were then compared to the data visualized within the HL2 device to ensure the data was being processed correctly. Additionally, a wrapper function from the CESSIPy library was called directly on the server to retrieve the estimated eigenfrequencies, damping ratios, and modal shapes of the model. The data was then compared with the HL2 visualizations to ensure accurate data transmission. Based on the results of this research, the following conclusions can be drawn:

(a) The program can accurately plot up to 18,000 points of acceleration data across three subplots in real-time without any noticeable performance drops. Within the AR environment, users can perform forward Fourier transforms with the collected experiment data at any point during the experiment. The results of these analyses are instantly stored and displayed on the HL2 device. This capability is pivotal for instantaneous data analysis and visualization in structural health monitoring.
(b) The application seamlessly communicates with an external Python module via HTTP requests, enabling it to perform SSI COV from recorded acceleration data. The Python module is hosted on a web development server and works in conjunction with PHP scripts to facilitate data transmission with the Unity application. In the future, a similar approach can be employed to establish communications with additional libraries, further expanding the capabilities of integrating AR with SHM.
(c) The system’s versatility extends beyond location constraints, as data transmission occurs seamlessly over Wi-Fi whether in the lab or field. If the server and HL2 are connected to the same network and the server scripts are updated to accommodate any network changes, users can access and use the system virtually anywhere.

The existing application development used in this research has certain limitations that should be considered for future enhancements. For example, as the number of plotted points increases or the experiment’s sampling frequency rises, the performance of the application on real hardware may be impacted negatively. In this research, the program was tested with 60 s of data across three sensors recording at 100 Hz, resulting in 6000 overall points per channel being plotted in real-time without any noticeable performance drops. While there is no set limit for plotting data in the application, substantially exceeding this threshold could result in a decreased frame rate and delayed script execution. As communication between the HL2 and the server is achieved through a wireless internet connection, the existing application is also constrained by the limitations of a wireless local area network. This may include range- or bandwidth-related limitations that could affect the performance of the program. When used in a new local area network, the internet protocol (IP) address referenced by the Unity application must be updated appropriately before the program can be used again. Finally, depending on the method of data collection, the parameters used in the SSI functions called from the external Python library may need to be manually adjusted for different experiments to ensure accurate mode shape estimations.

The advancements outlined in this research not only contribute to the accuracy of structural assessments but also significantly expedite data analysis while enhancing the overall safety of inspections. The proposed research holds the potential to revolutionize the field of structural health monitoring by introducing more efficient, data-driven, and real-time visualization. Future research endeavors will focus on testing with additional degrees of freedom and real-world case studies and expanding the scope of external modules that can be integrated with AR. This study is a fundamental stepping stone in SHM for real-time visualization of time-series data via AR. It paves the path to intelligent onsite visual inspections such as visualizing onsite modal estimates, characterizing structural damage, considering thermal and environmental correlations and influences, and tackling successive long-term variations of structural parameters.

## Figures and Tables

**Table 1:** PHP scripts used in the application.

Script	Function
showexperiment.php	Retrieves existing data from the MySQL database, if any, and echoes the result.
ssiexperiment.php	Sends existing data from the MySQL database to CESSIPy and echoes the output mode shape columns.
deleteexperiment.php	Deletes any existing data in the MySQL database so a new experiment can be performed.

**Table 2:** Time-domain visualization interface button functionalities.

Button	Function
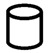 Get Data	Requests data from the MySQL database and plots it as a rendered line after storing it locally.
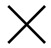 Clear Graphs	Clears the graph display.
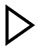 Start Capture	Starts an indefinite looping process where, approximately every 5 s, new data is requested from the MySQL database, and the graphs are automatically redrawn and scaled as necessary.
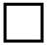 Stop Capture	Stops the live capture process.
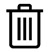 Delete Data	Deletes all data from the MySQL database so a new experiment can be performed.

**Table 3:** Frequency-domain visualization interface button functionalities.

Button	Function
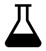 View FFT	Performs an FFT on the acceleration data plotted in the time-domain visualization window and plots the results.
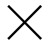 Clear Graphs	Clears the graph display.

**Table 4:** Stable mode shapes interface button functionalities.

Button	Function
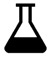 View SSI	Calls “ssiexperiment.php” to send experiment data to CESSIPy, returns and plots the resulting estimations.
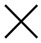 Clear Graphs	Clears the graph display.

## Data Availability

Data are contained within the article.
